# Contrastive learning-based multi-mechanism disentangled assessment for drug-drug interaction

**DOI:** 10.1186/s12859-025-06304-z

**Published:** 2025-11-27

**Authors:** Jinxiong Zhang, Yunjv Zeng, Chunyan Tang, Cheng Zhong, Hao Wen, Yang Liu

**Affiliations:** 1 School of Computer, Electronics and Information, Guangxi University, Nanning, China; 2 Key Laboratory of Parallel Distributed and Intelligent Computing in Guangxi Universities and Colleges, Nanning, China

**Keywords:** Risk identification, Contrastive learning, Disentangled representation learning, Mutual information, Link prediction

## Abstract

**Background:**

Polypharmacy’s ability to circumvent acquired resistance to single drug makes it a critical strategy for treating complex diseases. However, it inevitably carries risks of **d**rug-**d**rug **i**nteractions (**DDI**s) that may alter pharmacological activities and potentially lead to severe adverse events or mortality. Computational assessment of drug combination has emerged as an effective approach to support clinical decision-making. Current risk identification methods focus on mining historical interaction patterns to uncover underlying mechanisms, yet face challenges from data sparsity. While data augmentation strategy can mitigate such problem, conventional approaches often introduce noise that obscures core pharmacological mechanisms, undermining safety evaluation.

**Results:**

This study proposes a **M**ulti-**M**echanism **D**isentangled **D**rug-drug **I**nteraction assessment framework integrated contrastive learning, MMDDI, which includes two key components: (1) biologically-informed multi-view generation that creates high-quality augmented views, effectively addressing semantic distortion during data augmentation; (2) Mechanism-aware disentanglement that incorporates mutual information constraints to isolate interaction mechanisms from coupling of multi-modal and heterogeneous data, eliminating quantification bias. Contrastive learning integrates labeled and unlabeled data to enhance robustness against sparse observations.

**Conclusions:**

Comprehensive evaluations demonstrate that MMDDI with hit@4 of 0.86 outperforms the compared baselines, with ablation studies validating the critical contributions of multi-view contrastive and mechanism disentanglement. MMDDI continues to demonstrate excellent performance in cold-start scenarios, achieving accuracy of 0.94 and recall of 0.95. Clinically, MMDDI enables interpretable causal analysis of drug interaction pathways through its mechanism-aware representations, providing operability for optimizing therapeutic regimens.

**Supplementary Information:**

The online version contains supplementary material available at 10.1186/s12859-025-06304-z.

## Introduction

Polypharmacy has emerged as prevalent therapeutic strategy for managing complex or coexisting diseases due to their ability to alleviate resistance to single drug in patients (especially cancer patients). However, distinct substructures with chemical properties in drug may interact with each other, thus altering the drug efficacy, a situation that can increase risk of death [1]. Ideally, co-medication should be synergistic and toxicity-reducing, nevertheless, a review of previous literature revealed that interactions result in the majority of adverse reactions. Recent statistics from a hospital in the US showed that serious adverse drug reactions(ADRs) were found in 6.7% of hospitalized patients, with mortality rate 0.32% [2]. Thus, accurate evaluation of DDI risk is crucial for optimizing clinical therapeutic regimens [3]. However, detecting DDIs remains challenging, which traditional experimental methods are often costly and inefficient. It’s necessary to develop novel computational methods that can assist DDI prediction. In drug design and clinical diagnosis, risk identification aims to capture potential interactions by excavating information and patterns from the history of ADRs. In this light, the more DDIs we know, the better we can take effective measures to prevent ADRs. Early, data mining methods based on medical literature and clinical records [4–6], in which NLP techniques are mainly used to extract information about drug interactions, then parse trees and logic rules are used for prediction based on extracted interactions between new and existing drugs. The established logic rules are based on substructure similarity, yet many dissimilar drug pairs may still share common substructure unrelated to DDI [7]. With the shift towards structuring medical records, deep learning-based methods demonstrate significant efficacy in DDI prediction [8–10]. SSI-DDI [8] uses multilayer graph attention network (GAT) to efficiently extract drug substructures, then constructs substructure-substructure interaction SSI enabling detailed analysis of specific components of drug pairs, and predicts potential DDI. MSAN [11], from perspective of chemical structure altered by inducer/inhibitor, discards some substructures to simulate intensity of action between drug substructures. To some extent, the abovementioned methods optimize the screening and evaluating process of drug candidates.

These computational models can be divided into two categories: methods based on multi-source information fusion and those based on contrastive learning. A prominent approach within the first category integrates information from multiple scales [12, 13], modalities [14–17], and external knowledge bases [18], thus providing solution to the limitations of single-source data modeling in meeting modern accuracy requirements. MUFFIN [12] utilizes molecular structure of drugs and semantic information from knowledge graphs, respectively, to improve DDI prediction accuracy with multi-scale feature fusion. DDIMDL [10] ensembles DNN submodels based on drug SMILES, target, enzyme and pathway, and learns cross-modal representations of drug pairs to predict ADRs. DAS-DDI [17] further integrates drug molecular graph modal information, proposing a dual-view framework with drug association and drug structure. HetDDI [18] leverage rich structural information such as drugs and external biological knowledge to pre-train heterogeneous information network models. However, the constructed DDI graph based on limited labeled data is enormous and sparse, which greatly increases the computational complexity. And deep convolution can extract features while obscuring distinctions between individual features.

In contrastive learning-based DDI prediction methods, PHGL [14] applies three augmentation methods: atom masking, bond deletion and subgraph removal to drug molecular graph, which tailors a large mount of unlabeled data so that pretraining model to learn representative molecular structural features. Zhang et al. [19–21] performed **c**ontrastive **l**earning(**CL**) of learned drug local features and global features respectively. Nonetheless, such learning paradigm cannot essentially solve the sparsity of interaction data, and fails to fully explore the potential patterns of drug-drug interactions. Therefore, Lin et al. [13, 22, 23] employed CL for extremely unbalanced DDI types to significantly improve performance in predicting rare types with fewer samples. Utilizing stochastic edge partitioning, Wang et al. [15] decomposed the DDI graph into local subgraphs, where a graph encoder performed CL in the subgraph representation space to yield comprehensive drug embeddings.

However, several challenges still remain in existing studies. Drug representations obtained by supervised learning based on limited labeled data may be suboptimal. And multiple interaction mechanisms between drugs were not decoupled successfully [24]. It is hard or expensive to acquire data labels in many practical applications, whereas CL performs as a solution can improve model excellently with fewer labels. Experts in medicine have explained the internal mechanism of ADRs: drugs contain a mixture of constituents, chemical reactions such as redox, polymerization and oxidation will occur during the pharmaceutical process, the same is true for the DDI. Such as the co-medication in the patient’s body appeared in the single drug does not occur in the components, the toxic content of the medication is solubilized and lead to increased concentrations, P450 enzyme system is inhibited or induced so that the blood concentration changes, affecting the rate of metabolism and excretion eventually. DDI is mainly manifested in pharmacokinetics, which means that one drug alters the absorption, distribution, metabolism, and excretion of another drug, thus increasing or decreasing the blood concentration and target drug concentration. Related studies [25, 26] summarized three main mechanisms of drug action: transport, metabolism, and plasma protein binding processes, and explained in detail the evaluation methods of different mechanisms. Guan et al. [26] designed ADMET*-score* to evaluate drug-likeness of a compound by combining 18 drug properties involving five aspects including ADMET. Therefore, disentangling the different aspects of drugs’ action would help physicians to take timely precautions or examine patient’s status. DDIMDL modeled the operational principles ADRs as singular aspect, ignoring the fact that DDI is a result of more than one. When modeling highly coupled mechanisms, SSI-DDI assigns importance weights skewed from expectation, so that obscure dominant mechanisms and may mislead clinical decisions.

To address above-mentioned challenges, we proposes MMDDI, a multi-mechanism disentangled DDI assessment framework based on CL. First, MMDDI designs deep auto-encoding module that extracts information from both labeled and unlabeled data to embed these patterns into latent space. Specifically, two data augmentation operators with biological interpretation are proposed for creating different views to drug-pairs in CL module, which enrich the training data. To deal with the latter, we seek to clearly decouple DDI mechanisms. To be specific, mutual information is utilized as regularity of independence to encourage different aspects to learn information separate from each other. As such, it promotes encoder to capture semantic information well, providing interpretability for drug design and clinical consultation. Summarizing the main contributions of this paper as follows:

1. We propose MMDDI, a mechanism-aware joint medication risk assessor, whose multi-view contrastive learning module is equipped with biologically meaningful augmentation strategies and ultimately enhances model’s robustness.
2. We also develop specialized decoupling module for explicitly disentangling functioning mechanism responsible for occurrence of ADRs. It encourages specific mechanism to be able to learn characteristics from DDI samples independent of others, removing interpretability bias.
3. Experiments on two real datasets of different size and sparsity show that MMDDI obtains superior performance compared to baselines. Further ablation studies show that the benefit stems from the multi-view contrastive learning module and the decoupling module. In the additional DDI prediction task, MMDDI maintains outstanding performance in cold-start scenarios, achieving 93.85% accuracy and 94.96% recall.

## Discussion

### Contrastive learning

**Fig. 1:**
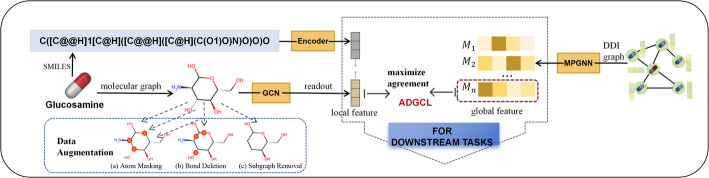
Main contrastive learning frameworks applied in DDI prediction

Earliest proposed in computer vision field, contrastive learning(CL) improves the number of samples in the training set by processing original image through flip, fog, and crop, each augmentation can transform data stochastically with some internal parameters (e.g. rotation degree, noise level). Further, these images with transformations are fed to train objective recognition model. Such data augmentation operators can improve robustness and generalization, and overcome model’s dependence on labeled data [27–29]. Li et al. [30–32] tackle data sparseness in sequential recommendation that predict the next item user may click on, introducing five operations(crop, mask, disrupt, substitution, and insertion), and randomly choosing two separate views to form positive pair. The principle of CL is that driving model to learn well characterized features by comparing the differences between views. Recently, inspired by self-supervised learning, a series of studies have emerged to apply CL to learn enhanced drug-pair representations in DDI prediction tasks with limited labeled data. CL consists of two primary components: data augmentation for boosting training data and contrastive loss used as self-supervised signal. Based on this, as illustrated in Fig. 1, Yuan et al. [14] proposed three augmentation strategies for molecular graph. Zhuang et al.(2023) [33] assumed that local and global features share similar semantic and proposed ADGCL that utilizes graphCL to maximize agreement between local and global representation of drug molecules, while the irrelevant semantic is minimized, as well as incorporates microsupervised learning to mitigate category imbalance. MDDI-SCL [13] encodes multi-omics similarity and takes CL in self-supervised manner to embed samples of the same type closer to each other in the latent space, which promotes performance in more fine-grained fashion. DSN-DDI [9] introduces a novel multi-view drug substructure framework that learns substructures from individual drug views and updates drug representations from drug-pair views. Whereas the practice allows for in-depth understanding of drug properties as well as drug interactions, deep graph convolution operation blurs differences between substructures. Although CL reveals new prospects, the technical perspective of augmentation has not been considered in drug interaction studies. In a recent study, Wang et al. [15] decomposes the DDI graph into multiple local subgraphs via stochastic edge partitioning and performs CL in the latent space. Although this approach reduces the computational complexity, it lacks semantic support and fails to address the performance degradation in inductive setting. MSAN [11] proposed substructure discarding with consideration that substructure changes may affect drug interactions and simulated the strength of interactions between substructures of two drugs based on similarity. The method provides an effective and intuitive way to understand DDI by directly incorporating the molecular structure of drugs. Departing from standard practice, this paper proposes two augmentation strategies, flip and shuffle, for overcoming the limitation that previous practice is difficult to retain core semantics, and ensuring interpretability of the augmented view.

## Decoupled representation learning

**Fig. 2:**
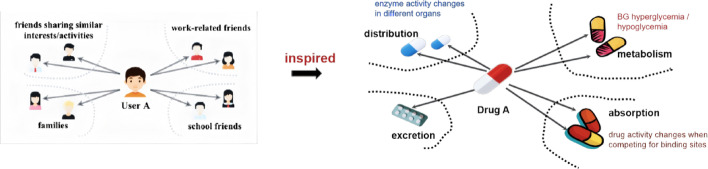
Toy example of multi-facet mechanisms behind drug-drug interaction network

In the field of recommendation systems, scholars conduct recommendation algorithm research based on user-item historical interaction. It is widely believed that multiple underlying motivations are coupled behind the user’s behavior. Ma et al. [34] proposed MacridVAE, the first attempt to incorporate decoupled representation learning into behavioral history of users at macro and micro levels. Subsequently, sequence-to-sequence training strategy was proposed to extract additional supervised learning signals in the uncoupled potential space [35–37]. Sha et al. [36, 38, 39] argued that users make social connections with others and consume items with different motivations, which reflect user’s subjective interest in carrying out the interaction, e.g., user may interact with an item because of its specifications, layout, and shelf-expiration date, and so forth. When user wants to buy bread, shelf life may be main motivation, i.e., different motivations contribute differently to item recommendation. Therefore, we define disentanglement as detaching the integral mechanisms/motivations behind a variable into combinations of multiple independent variables, and similarly for multi-variable linear regression, which also requires that variables should be isolated from each other. Consistent with this paradigm, similarly, most DDI prediction and co-administration risk identification predominantly leverage drug-drug interaction or drug-substructure co-occurrence matrices, and existing works ignore the fact that the occurrence of ADRs may be a consequence of multiple underlying mechanisms [24, 40]. For example, some chemicals may exhibit diverse mechanisms depending on their structures, Hao et al. [41] addressed this issue by developing MeTDDI, an approach that is capable of probing the main mechanisms of enzyme inhibition of chemicals, which can aid medicinal chemists in identifying metabolic sites and thus better understanding DDI’ occurring. As shown in Fig. 2, drug A may potentially have adverse effects by affecting the absorption, distribution, metabolism and excretion process of drug B. Joint use of drugs is risky for reasons that glycemic changes during metabolism, distributional changes of drugs due to changes in enzyme activities in different organs, and the risk of changes in the activity of drugs competing for binding sites during absorption [24], etc. In addition, the dose, frequency and delivery route of administration may also influence DDI, leading to safety concerns. According to Peng et al. Peng et al. [25], CYPs-mediated metabolism and transporters-regulated cellular uptake constitute the dominant biological pathways underpinning clinically significant DDIs Highly engangled is detrimental to the interpretability of drug design and physician practice diagnosis. For example, assume that DDI mechanism is decoupled into metabolism$D_{m}$ and absorption$D_{a}$ :

1 $$ \begin{gathered} D_{a}^{{\prime \:}} = 0.6D_{a} + 0.4D_{m} ,D_{m}^{\prime } = 0.4D_{a} + 0.6D_{m} , \hfill \\ D_{{fuse}}^{\prime } = 0.8D_{a}^{{\prime \:}} + 0.2D_{m}^{{\prime \:}} = 0.56D_{a} + 0.44D_{m} , \hfill \\ \end{gathered} $$

Where$\:{\:D}_{m}^{{\prime\:}}$ and $\:{D}_{a}^{{\prime\:}}$ denote the uncoupled representation, respectively. The final fusion weights of 0.56 and 0.44 are significantly different from expected 0.8 and 0.2. Therefore, dissociation enhances interpretability, and clarifying the dominant mechanism is more informative for clinicians’ decision-making.

Accordingly, the most direct guidance of disentangling at pharmacodynamic mechanisms in conjunction with multi-omics drug profiles is that we attempt to disentangle ADRs rather than aggregation. This approach clarifies dominant clarifying the dominant pharmacological aspect and eliminates interpretability bias inherent in prior studies, thereby preventing misrepresentation of co-administration safety .

## Methods

### Problem formulation

In our task, each drug is encoded with functional substructures derived from SMILES using RDKit [42]. The dataset for this study consists of the following three sets: **(a)** a set of SMILES-formatted strings ***S***, which is used to generate drug-embedding tables $$ E_{1} = \left[ {e_{1} , \ldots ,e_{i} , \ldots ,e_{n} } \right] $$ and $$ E_{2} = \left[ {e_{1}^{\prime } , \ldots ,e_{i}^{\prime } , \ldots ,e_{n}^{\prime } } \right] $$, $$ e_{i} $$ denotes drug *i* similarity features, and$$ e_{i}^{\prime } \in \left\{0,1\right\}^{d} $$is a multi-hot vector and represents the substructure composition of drug *i*, the *h*-th item of $$ e_{i}^{\prime } $$is 1 if drug *i* contains substructure *h*, *i*=1,…,*n*, *h*=1,…,*d*, where *n* is the total number of drugs and *d* is the total number of RDKit-derived substructures; **(b)** a drug pair set of known drug-drug interactions *I*={(*DrugA*, *DrugB*)|*DrugA*, *DrugB*$$ \in S $$}, $$ m = |I| $$; **(c)** a set of drug pairs ***F***=(***S***$$ \times $$***S***)\*I*, which consists of drug pairs whose interactions have not been reported, $$\tau = |F| $$. In other words, known DDIs in the dataset are considered as positive samples, while unknown DDIs are treated as negative samples. Thus, for a given drug *DrugA*, $$ S_{{DrugA}}^{ + } $$ is defined as the set of drugs interacting with *DrugA*, oppositely, $$S_{{DrugA}}^{-} $$ is defined as the set of drugs without known interactions with *DrugA*. Unlike determining whether there exists interaction between drugs, co-medication risk identification is primarily based on drug-pair attributes, utilizing labeled and unlabeled data to constitute triple set $$ D = < DrugA,DrugB_{i} ,DrugB_{j} > DrugB_{i} \in S_{{DrugA}}^{ + } , $$   

$DrugB_{j} \in S_{{DrugA}}^{-}$. Thereby, the sampled drug pair $(DrugA,\:DrugB_{i} ) \in I$ is called as positive sample, and the sampled drug pair $(DrugA,\:DrugB_{j} ) \in F$ is called as negative sample. A candidate drug needs to be evaluated for its potential to be a perpetrator (inhibits or induces enzymes or transporters) or victim (whose pharmacokinetic is changed by a perpetrator) of DDIs with likely co-administered drugs. According to the input drug, the system outputs: (i) a prioritized list of high-risk DDIs with (ii) associated risk quantification coefficients, thereby supporting evidence-based avoidance strategies. The mathematical formulation is defined as Eq. (2). The output of ${\text{Prob}}(\cdot)$ serves as a risk quantification coefficient, typically implemented by sigmoid function that maps the input to the probability space.

2 $$ {\text{argsort}}\left( {{\text{Prob}}\left( {\left. {DrugB \in F_{{DrugA}} } \right|\left\langle {DrugA,DrugB_{i} ,DrugB_{j} } \right\rangle } \right)} \right){\text{ }}[:topk], $$

## Drug association encoder and drug-pair encoder

It is well known that drugs with similar chemical structures may exhibit similar activities and that similarity potentially symbolize valid information about DDI. Furthermore, similarity provides richer context for each drug, which helps to capture more complex inter-drug relationships [13, 17]. Inspired by this, we use multi-hot encoding to express drug attribute composition regarding various distinct attribute categories, then design drug multi-attribute features to help the model extract latent cross-drug associations. Unlike post-fusion adopted by DDIMDL, we concatenates all features as unified DNN inputs [43], which has been proved to be an effective behavior by Deng et al. [10]. Subsequent processing employs a position-embedding-free transformer encoder(comprising two sub-layers) to exhaustively distill information embedded within the feature vectors. The integrated multi-head attention (MHA) mechanism explicitly models dependencies between drug pairs. The $Tanimoto$ [44] coefficient is calculated by Eq. (3) to measure attribute composition similarity between drugs.

3 $${Tanimoto}(S_{i} ,\:S_{j} )^{{(\omega )}} = \frac{{|\:S_{i}^{{(\omega )}} \cap S_{j}^{{(\omega )}} |}}{{|S_{i}^{{(\omega )}} \cup S_{j}^{{(\omega )}} |}} $$

Where $\omega \in \{ substructure,\:target,\:enzyme,\:pathway\} \:$denotes attribute category, $S_{i}^{{(\omega )}}$ and $S_{j}^{{(\omega )}}$ represent the attribute composition vector of $\:{Drug}_{i}$ and $\:{Drug}_{j}$ in terms of attribute category $\:\:\omega\:$ respectively. $\left| {S_{i}^{{(\omega )}} \cap S_{j}^{{(\omega )}} } \right|$ is the intersection size of $\:{Drug}_{i}$ and $\:{Drug}_{j}$ attribute composition vectors, $\left| {S_{i}^{{(\omega )}} \cup S_{j}^{{(\omega )}} } \right|$ denotes the union size of $Drug_{i}$and $Drug_{j}$ attribute composition vectors. In essence, ${\text{Tanimoto}}(S_{i} ,S_{j} )^{{(\omega )}}$ indicates Intersection over Union of $\:{Drug}_{i}$ and $\:{Drug}_{j}$ attribute composition in terms of attribute category $\:\text{ω}$. Four attribute composition similarities between $\:{Drug}_{i}$ and $\:{Drug}_{j}$ are concatenated horizontally to form initial drug-pair embedding. The initial drug-pair embedding is then fed separately into two-layer auto-encoder. Therefore, MMDDI is allowed to extract information from both labeled and unlabeled drug pairs and embed these patterns into latent space.

4 $$z = \text{F}^{{(l)}} (\text{BN}(\sigma(W_{e} x))),\text{ }\widehat{x} = \text{F}^{{(l)}} (\text{BN}(\sigma(W_{d} z))), $$

5 $$ \mathcalligra{L}_{{re}} (x,\widehat{x}) = \frac{1}{d}\sum\nolimits_{{i = 1}}^{d} {\left( {x_{i} ,\:\widehat{x}_{i} } \right)^{2} }  $$

In encoder of CL module, dependencies among drug attribute categories are fully captured with MHA, drug pair interaction patterns are projected into the same latent space to manifest different contributions of substructures and extract the dependencies between frequent substructures. As illustrated in Eq. (4), $x \in {\mathbb{R}}^{{n \times d}}$is constructed by $Concat(e^{s} ,e^{t} ,e^{e} ,e^{p} )$, $W_{e} \in {\mathbb{R}}^{{k \times d}} \;{\text{and}}\;W_{d} \in {\mathbb{R}}^{{d \times k}} \:(d\ll k)$ are trainable weight matrices, BN [45] denotes batch normalization, which solves Internal Covariate Shift within model, and was shown to improve performance when added to network layers. DDI is mainly caused by few functional substructures, while the rest is less relevant. Therefore, feature compression is used to remove redundant information, minimizing Eq. (5) to ensure that important information was maximally restored and alleviate memory pressure.

**Fig. 3:**
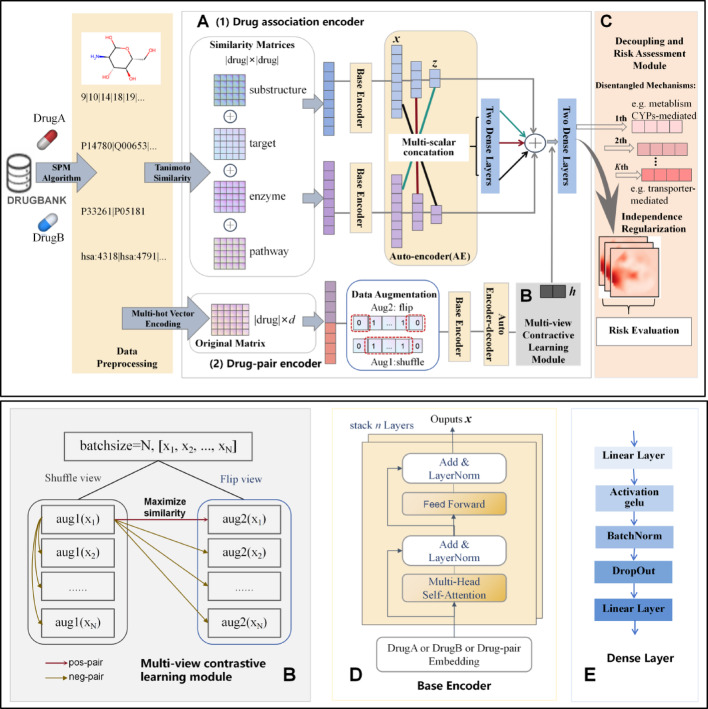
Framework of MMDDI model. MMDDI comprises five main components: (**A**) The drug encoding module, which corresponds to two entrances of the model, including drug association encoder and drug-pair encoder. The former encodes similarities of the specific drug to other drugs as features, while the latter is multi-hot encoding, with index values of 0 and 1 used to indicate whether the drug molecule contains the substructure or not. (**B**) The multi-view contrastive learning module incorporates flip and shuffle augmentation operators to generate biologically coherent views. Schematic illustrations further detail the embedding alignment process between positive sample pairs and negative counterparts. (**C**) The decoupling and risk assessment module, disentangles DDI mechanism into $\:\text{K}$ discrete pharmacological aspects, which are then fused to output risk value through multi-head attention. (**D**) Base encoder, which employ two sub-layer of transformer structure but without positional encoding. (**E**) Dense layer.

### Multi-view contrastive learning module

Current research on DDI based on CL not consider augmentation from technical view, so this paper introduces two biological simulation strategies, flip and shuffle, for constructing positive pairs, so as to overcome the difficulties in retaining core semantics. The SPM [7] algorithm was used to extract frequent substructures from SMILES, encoding them into fixed-dimensional vectors. This section starts with how to construct the augmented view and how to definite the positive and negative pairs, followed by the contrastive loss function.

- (a) Flip. Flip 0 to 1, which means that DDIs change the substructure contained in the drug. $x = [v_{1} ,v_{2} ,...,v_{n} ],v_{i} \in \left\{0,1\right\}$if $\mathop \sum \limits_{{k = 1}}^{n} 1~[v_{k} = 0] \ge n\alpha$, Then $n\upalpha\:$ is taken randomly from the index that takes value 0 and flipped to 1, where $\:{\upalpha\:}$ is hyper-parameter named flip rate. We assume that DDI involves chemical reaction that produces new substance. Such strategy could improve accuracy and recall rate of risk identification theoretically, because the enhanced view indicates that the drug possesses new function groups, which increase the likelihood of adverse effects occurring.
- (b) Shuffle. Randomly selecting the starting index in drug vector and shuffling the segments with consecutive lengths of $n\upalpha\:$. The operation is useful for removing interference from feature alignment by ensuring total count of 1 remains unchanged. Sample $\:{k}$ from $[1,~2,~...,~n - n\alpha + 1]$as start index,$~shuffle([k,~k + n\alpha - 1])$ [18]. utilized transformer without positional encoding, which does not fully consider randomness of the substructure arrangement. As a result, the view created by shuffle enhances robustness to different permutations of substructures that combines the bidirectional advantages of the BiRNN-DDI [16].

Augmentation strategies provide more opportunities for the non-popular drug-pairs to learn. The augmented vectors are further embedded into low dimension for comparison via encoder-decoder, and the *InfoNCE* Loss [28] is minimized to maximize the mutual information(MI) between the positive sample pairs. Specifically, *MI*, illustrated by Eq. (6), is defined to measure interdependence between random variables. It measures the degree to which the uncertainty of *Y* is mitigated given the information *X*, which is ultimately transformed into KL divergence between the product of the joint distribution and its marginal distribution. Here, *X* and *Y* represent the overall variables of the outputs from the two augmented views.

6 $$\: \begin{aligned} MI(X,Y) = & H(Y) - H(Y|X) = \sum\nolimits_{{x,y}} {p(x,y)} \log \frac{{p(x,y)}}{{p(x)p(y)}} = D_{{KL}} \left( {\left. {p(x,y)} \right\|p(x,y)} \right) \\ \end{aligned} $$

Contrastive learning loss applied as objective function to maximize the consistency of anchor nodes with positive instances, and minimize the similarity of anchor nodes to negative samples. The last layer of CL uses ReLU activation function, ReLU produces sparse representations, which further facilitates easier interpretation of the codes.

7 $$\: \begin{aligned}   \mathcalligra{L}_{{InfoNCE}}  =  &  - \log \frac{{\exp (sim(q,x^{ + } ))}}{{\sum\limits_{{i = 0}}^{N} {\exp (sim(q,x_{i} ))} }} \\     &  =  - \log \frac{{\exp (\log p(x^{ + } ,q))}}{{\sum\limits_{{i = 0}}^{N} {\exp (\log p(x_{i} ,q))} }} \\     &  =  - \log \frac{{p(x^{ + } ,q)}}{{\sum\nolimits_{{i = 0}}^{N} {p(x_{i} ,q)} }} \\  \end{aligned}  $$

8 $$ sim(q,x) =  - E(x,q) = \log p(x,q) $$

Hypothesized that positive sample pairs $$ p(x^{ + } ,\:q) $$ are from the joint distribution, while the negative samples are independent samples based on the marginal distribution $$p(x^{-} ) $$ and $$\ p(q) $$[28,46]. Equation (6) optimizes the MI between the positive and negative by maximizing the joint probability of positive sample and minimizing edge probability of negative sample, which is refined by Eqs. (8–9), and Fig. 3 shows more details of the process.

9 $$\sum\nolimits_{{i = 0}}^{N} {p(x_{i} ,q) \approx p(x^{ + } ,q) + N\cdotp(x^{-} )p(q)} $$

10 $$ \begin{aligned}   \mathcalligra{L}_{{InfoNCE}}  \approx  &  - \log \frac{{p(x^{ + } ,q)}}{{N\cdotp(x^{0} )p(q)}} \\     &  \approx  - E_{{p(x,q)}} \left[ {\log \frac{{p(x,q)}}{{p(x)p(q)}}} \right] \\  \end{aligned}  $$

Finally, in the specific implementation, the loss is transformed into the form of Eq. (10), where both $h_{i}$ and $h_{j}$ represent the latent representations of drug-pair output by the CL module.

11 $$  {\mathcalligra{L}}_{{InfoNCE}}  =  - \log \left( {\frac{{\exp (h_{i}  \cdot h_{j} /t)}}{{\sum\nolimits_{{h^{0}  \in H_{i}^{ - } }} {\exp (h_{i} *h^{0} /\tau )}  + \sum\nolimits_{{h^{ + }  \in H_{i}^{ + } }} {\exp (h_{i}  \cdot h^{ + } /\tau } )}}} \right)   $$

## Decoupling and risk assessment module

Microscopically, reactions between two drugs usually involve interactions between their substructures, which are closely related to their biological activities. And it helps in potential risk assessment to understand the mechanism of action when two drugs are combined and clearly unpacked at macro. Thus, we need to associate interaction mechanism meaning to each disentangled module. The CYPs, which constitute the major enzyme family capable of catalyzing oxidative biotransformation, are of high clinical relevance for DDIs. The high permeability drugs, mainly eliminated by metabolism, are often involved in CYPs-mediated DDIs. The poorly permeable drugs, predominantly eliminated by renal and/or biliary excretion, often suffer from transporter-mediated DDIs. For different decoupled aspects, their contributions are assigned through MHA. However, previous studies [3] for the decomposition of the mechanism are accomplished by simple segmentation through neural networks without adding explicitly independent regularity, resulting in biased interpretability.

Therefore, in our decoupling module and risk assessment module, KL divergence is applied as regularization to ensure different action features $p$ and $q$ derived from decoupling are independent of each other. $K$ is the number of aspects.

12 $$\begin{aligned} D_{{KL}} (p,\:q) = & \sum\nolimits_{i} {p(x_{i} )} (\log p(x_{i} ) - \log q(x_{i} ) )= E_{{x\sim p}} \left( {\log \frac{p}{q}} \right) \geq 0 \\ \end{aligned} $$

MMDDI maps each input sample to risk value, risk probability scores of positive and negtive samples used to calculate BPR pairwise loss, which backfires to update model parameters. BPRloss is commonly implemented for binary preference ranking, in particular for processing implicit data, where the goal is to maximize the log-likelihood of the difference in scores of the positive samples relative to the negative samples:

13 $$ Loss_{{bpr}}  =  - \frac{1}{m}\sum\nolimits_{{k = 1}}^{m} {\log s(\widehat{r}_{{(k,\:i)?I}} \: - \:\widehat{r}_{{(k,\:j)?F}} )}  $$

$\sigma(x)$ is the sigmoid function that maps the score difference to [0,1], which in turn maximizes the joint probability across samples through the negative log-likelihood. The idea of applying BPR modeling in this paper is that for any $DrugA$, the interaction $DrugB$ corresponding to $DrugA$ was labeled, and if $DrugA$ acts on $DrugB_{i}$when both$DrugB_{i}$and $DrugB_{j}$are available, then triples $\left\langle {DrugA,\:DrugB_{i} } \right\rangle$ are obtained, which indicates the level of risk associated with $\left\langle {DrugA,\:DrugB_{i} } \right\rangle$ conjunction is higher than $\left\langle {DrugA,\:DrugB_{j} } \right\rangle$. $t$ such triples represent $t$ training samples in total.    

## Joint training objective

Following the most common multi-task training strategy, we had the overall loss function as follows:

14 $$  \mathcalligra{L}_{{total}}  = \alpha  \mathcalligra{L}_{{re}}  + \beta  \mathcalligra{L}_{{InfoNCE}}  + \gamma  \mathcalligra{L}_{{ind}}  +  \mathcalligra{L}_{{bpr}}  $$

### Experiments

#### Datasets

A clinically relevant DDI may occur when the perpetrator affects one of the main pathway of the victim drug. DDIs arise from diverse mechanisms, including enzyme inhibition/induction, transporter-mediated processes, and substructure interactions [25]. Given their comprehensive information coverage of these mechanisms demonstrated in prior research, Dataset1 [10] and Dataset2 [22] were selected as benchmark datasets for this study. Table 1 shows the details of the datasets. And both datasets provide only positive samples, negative samples are selected following the leave-one-out method in SRR-DDI [14, 17, 47] model. We generate negative counterparts by sampling complement set of positive drug pairs set in model training, while utilizing one-to-many strategy for more negative samples played as candidates in validation phrase.

**Table 1:** Summary of datasets used in our experiments

Name	Drugs	Interactions	Sparsity	Feature types
^a^Dataset1 [10]	572	37,264	0.8825	SMILES, target, enzyme, pathway
^b^Dataset2 [22]	1258	323,539	0.7885	SMILES, target, enzyme

^a^The Dataset1 can be downloaded from https://github.com/ShenggengLin/MDF-SA-DDI/blob/main/event.zip.

^b^ The Dataset2 can be accessed at https://github.com/ShenggengLin/MDF-SA-DDI/blob/main/Dataset2_drug_information.zip.

**Fig. 4:**
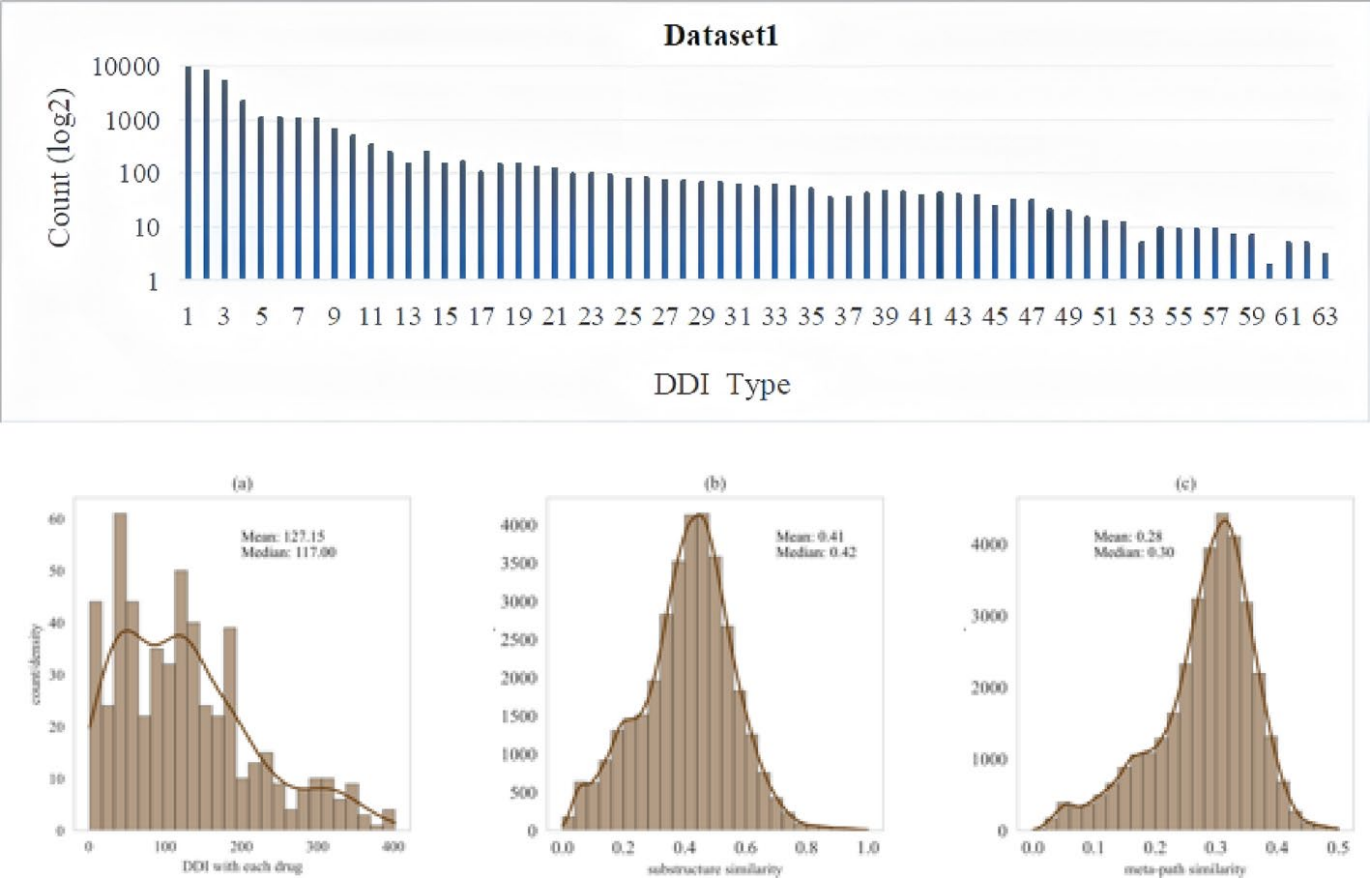
Descriptive statistical analysis of Dataset1

Pre-processing the above datasets so that they are suitable for baselines. Drugs and corresponding DDIs for which SMILES cannot be found from pubchem [48] will be excluded, single atom drugs are also removed. Finally, 540 and 1175 drugs, 34,268 and 292,046 DDIs for Dataset1 and Dataset2 were obtained, respectively. The number of drugs in dataset2 are approximately twice that of dataset1, which can validate whether MMDDI is scalable. Dataset1 was statistically analyzed and visualized as shown in Fig. 4. Each drug interacted with 127 drugs on average, or 23.5%, and the short-head-long-tail distribution of DDIs suggests presence of both prevalent and cold-start drugs, which is particularly important for assessing the risk of co-administration. Structural and meta-path similarity scores between drugs were generally low, with slightly left-skewed. It indicates that limited information characterized by similarity encoder, so additional encoders are necessary.

### Metrics

In this paper, risk assessment for co-medication is performed in order to avoid potentially risky prescriptions, which is modeled as a top-K avoidance scenario, and hence the following metrics are applied according to user-item interaction in recommendation systems [34–36, 38, 39].

HIT@K, serves as the top-K hit ratio metric, preserving its rank-sensitive accuracy semantics. $DrugA$ in the test set of drug pairs as anchor, employing leave-one-out [49] to generate candidates as $DrugB$(i.e., one positive and randomly sampled *N* negative examples). The anchor is assigned an accuracy score of 1 if the positive instance ranks among the top-K predictions.

15 $$ ACC@K = \frac{1}{{N_{A} }}\sum\nolimits_{{DrugA}} {[1]_{{DrugB_{{pos}} \in \arg sort(scores)[:K]}} } , $$

16 $$ NDCG@K{\text{ }} = {\text{ }}\frac{1}{{N_{A} }}\sum\nolimits_{{DrugA}} {\sum\nolimits_{{i{\text{ }} = {\text{ }}0}}^{{K{\text{ }} - {\text{ }}1}} {\frac{{r_{i} }}{{\log _{2} (i{\text{ }} + {\text{ }}2)}}} } , $$

17 $${\mathrm\:{MRR@K=}\text{ }\frac{\mathrm{1}}{N_A}\sum_{DrugA}{\frac{\mathrm{1}}{{{rank}_{pos}}\mathrm{+1}}\mathrm{\text{ .}}}}$$

To further reflect whether positive examples of $$ DrugB $$ with higher risk values appear at ahead, NDCG@K(Normalized Discounted Cumulative Gain) and MRR@K(Mean Reciprocal Rank) are introduced. Where $$ r_{i} $$ denotes the relevance to $$DrugA $$, for simplicity, we set the relevance of positive examples to 1, otherwise to 0. $$ rank_{{pos}} $$ denotes rank index of in all candidates, and the lower the rank, the smaller the value, and the value of 0 when it is not in top-K list.

When physicians or patients use co-medication prescription that has not been historically documented, MMDDI is able to assess risk of the prescription. This will assist physician to clarify the mechanism of action, ultimately determining whether the prescription is feasible and deciding intake dosage. For comparison, this article will show case study of drugs that potentially risky in combination with Fenfluramine.

### Training implementation

**Fig. 5:**
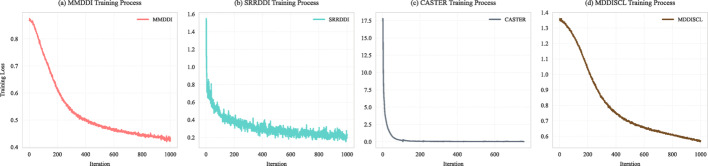
Training loss curves for MMDDI and baselines

Due to large number of negative samples, it is necessary to randomly sample the same number of positive samples to construct triples for training. Training process utilizes BGD from batchsize setting $[32,~64,~128]$, and sets $epoch = 15$. Meanwhile, CL temperature parameter $T = 0.05$, learning rate $lr = 0.00002$, and the number of disentangling aspects $D_{K} = 2$ were set empirically for simplicity. Drop rate set to 0.4 and we apply 5-fold cross-validation to DDIs instead of drugs, which ensure generalization and reduce randomness caused by data division. Early stopping strategy is also applied. And all baselines follow original configuration. Figure 5 illustrates the training loss of some baselines varying with the number of backpropagation iterations. Overall, CASTER converges rapidly, MMDDI changes more smoothly, and SRRDDI suffers from fluctuations locally, but all of them tend to converge in the end.

### Baselines

We compared MMDDI to baselines that were dealing with drug substructures and similarities, or had CL modules, respectively.

**VanillaCF**: The most primitive collaborative filtering(CF) algorithm with no specific information about drugs. Recommendations are made by medication co-occurrence matrix. But it cannot cope with drug cold start.

**Similar-based** [50]: Using similarity-based matrix heuristics. According to [35] it can be divided into network similarity-based method and network embedding-based method [10]. Network similarity-based method directly uses connections between drugs to calculate similarity, common similarity metrics include common neighbors, meta-paths, Jaccard coefficient, etc. Network embedding-based method maps drugs in the network to low-dimension by calculating cosine similarity. The former is suitable for simple tasks, while the latter is able to capture more complex structural information.

**PathSim1 & PathSim4** [51]: The former CF is based on the metapath similarity of substructures and is able to elaborate DDIs through substructure pairing. The latter is based on metapath similarity of multi-omics.

**NetworkSim** [52]: Network similarity-based approach, is capable of capturing higher-order similarity, inferring potential DDIs through label propagation.

**NetEmb** [10]: The nodes in drug homogeneous network are mapped into low-dimensional embeddings by matrix decomposition. We employ DNNs to perform DDI risk assessment, using regular terms to constrain the low-dimensional embeddings of same drug in drugA matrix and drugB matrix to be as close as possible.

**LR1 & LR4** [53, 54]: Corresponding to the two inputs of MMDDI, the former uses drug structure similarity as features to predict DDI using logistic regression, while the latter uses multi-omics.

**DDIMDL** [10]: Four types of drug features were used separately to construct DNN sub-models to learn cross-modal representation.

**CASTER** [7]: Functional substructures that play major role are mined by SPM to effectively characterize drug. Additionally, a dictionary learning module that measures the relevance of each input substructure to the DDI results and improves interpretability of the predictions.

**MDDI-SCL** [13]: Adverse events are predicted based on multi-omics similarity profile. We modified the last layer of original model setup from multi-class to single-classification that can be seen as regression.

**SRR-DDI** [3]: Substructure refinement mechanism is proposed, and also utilizing multi-scale fusion.

MMDDI learns interpretable representations, is able to offer more than just improved performance. Since metrics used in this paper are different from original article, to ensure consistency, we change the output layer of baselines to one neuron, and all other settings follow the original.

## Results

Overall performance are presented in Table 2. The results on Dataset1 show that MMDDI obtains the optimal performance in hit rate, and the other metrics are only second to NetSim, the performance of SRRDDI based on 2D molecular structure is close to that of MMDDI, reflecting that effectiveness of mechanism disentangling in capturing the critical pathways of action. Among non-NN approaches, algorithms such as VanillaCF and NetSim, which are based only on implicit feedback of drug interactions, obtain comparable performance. Even, we found that VanillaCF performs much better than other similarity-based deep learning methods. The reason may be that structural similarity and meta-path similarity scores between interacting drugs are generally low, as shown in Fig. 4(Section Experiments), both of them are slightly left-skewed, which are not representative by encoding them as input to the neural network. VanillaCF, originally developed for recommendation systems, operates based on drug interaction co-occurrence matrices and excels at recommending popular drug combinations. In e-commerce item recommendations, this often results in excessive homogenization, failing to meet consumers’ personalized needs. In contrast, the augmentation strategies in MMDDI provide more learning opportunities for non-popular drug pairs. However, VanillaCF also has limitations. For instance, while digoxin interacts with both estrogens and thyroid preparations, VanillaCF would infer that estrogens and thyroid preparations should also interact based on the co-occurrence matrix, despite their actual pharmacological compatibility.

**Table 2:** Experimental results. The highest value in each column is shown in bold. The italic values are the second-best performance baselines for each metric

	Dataset1			Dataset2		
	HIT@4	NDCG@4	MRR@4	HIT@4	NDCG@4	MRR@4
**VanillaCF**	*84.98*	*72.82*	*68.67*	**85.05**	**69.01**	**63.57**
**PathSim1**	35.73	25.24	21.73	73.70	56.74	51.01
**PathSim4**	30.81	21.21	18.00	75.24	60.22	55.14
**NetSim**	81.27	**74.54**	**72.23**	*79.76*	*66.37*	*61.84*
**NetEmb**	66.33	46.15	39.41	60.44	38.72	31.48
**LR1**	79.19	54.53	46.29	56.48	37.12	30.59
**LR2**	71.18	49.05	41.64	58.85	39.24	32.55
**DDIMDL**	50.21	29.85	23.11	52.76	32.45	25.72
**MDDISCL**	47.92	28.73	22.38	33.16	17.67	12.64
**CASTER**	57.58	37.24	30.47	57.05	36.45	29.60
**SRRDDI**	76.71	55.88	48.88	71.03	48.69	41.22
**MMDDI**	**86.12**	55.43	45.08	74.20	45.82	36.37

Furthermore, we observe that using all features does not lead to better results than single feature type. Results of PathSim1 and PathSim4, LR1 and LR2 illustrate that it is not the case that more detailed information improves the ability to assess risk. NN was named as black-box model, it is obvious that the more detailed information is more favorable, and the collaborative filtering algorithm fails to reflect that advantage. LR1 and LR2 validate the effectiveness of drug similarity encoding in network prediction.

In Dataset2, the performance of baselines fused with NN all suffered decreases from different degrees, which we speculate stems from datasets’ sparsity. We split datasets into training and test based on interactions with 4:1. Both MDDISCL and MMDDI have CL module to mitigate data sparsity, and thus can show their advantages in dataset1. Despite enhanced performance in dataset2, CF algorithms are unable to handle cold-start scenarios, which greatly limits development of new drugs. Algorithms such as MMDDI can effectively avoid this shortcoming by designing a series of biological simulation processes to improve interpretability, but at the cost of performance degradation. Therefore, design of biological simulation applicable to SRRDDI or further optimization of MMDDI is needed to improve model robustness.

### Ablation study

- To verify the impact of each component in MMDDI on its performance, five variants were designed for ablation experiments on dataset1 :
- ***encoder & Fusion***(*Vanilla*): the vanilla baseline of MMDDI, consists of only the drug encoding module and the fusion module. The fusion module concatenates the multilayer outputs of encoders at different scales.
- ***Con***: This variant is designed to explore performance when only CL module exists.
- ***Con & De***: Since MMDDI contains two entries, we examine the performance when containing the multi-view CL and decoupling module simultaneously.
- **MMDDI** ***w/o De***: In this variant the decoupling module is removed or can be seen as $D_{K} = 1$, similar to MDF-SA-DDI [22].
- **MMDDI** ***w/o Con***: Only CL module is removed.

**Fig. 6:**
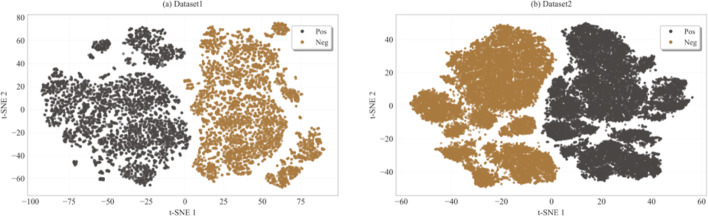
Visualization of positive and negative examples in latent space

The performance of MMDDI and its variants on dataset1 is summarized in Table 3. Firstly, risk identification of drug pairs can be improved by encoding similarity, as a result *De & Con* without that encoders faced declining. *Con* with multi-omics feature encoding of drug-pairs better learns interaction mechanism between similar chemical substructures, and two augmentation strategies simulate this process in biological sense, with significant enhancement for improving risk warning. Further, T-SNE was employed to visualize the learned low-dimensional embeddings of samples in 2D, as shown in Fig. 6, which indicates that with CL and BPR pairwise loss exhibits excellent ability in differentiating potential DDIs. In contrast, the decoupling module *De* contributes interpretability to MMDDI, with sacrifice in performance, but within acceptable levels.

**Table 3:** Results of ablation experiments

	HIT@4	NDCG@4	MRR@4
***Vanilla(w/o Con & De)***	73.16	51.50	44.23
***MMDDI w/o Con***	20.56	14.57	12.53
***MMDDI w/o De***	77.53	59.27	53.14
***Con***	84.94	55.40	**45.49**
***Con &De***	43.73	24.67	18.29
**MMDDI**	**86.12**	**55.43**	45.08

In short, each module’s changes and mechanisms affect the overall performance. Design of MMDDI, macroscopically carefully refining drug association features and microscopically considering potential substructure shifts, and then disentangling the mechanisms is crucial for recognizing potential DDIs and assisted decision making.

### Parameter sensitivity analysis

**Fig. 7:**
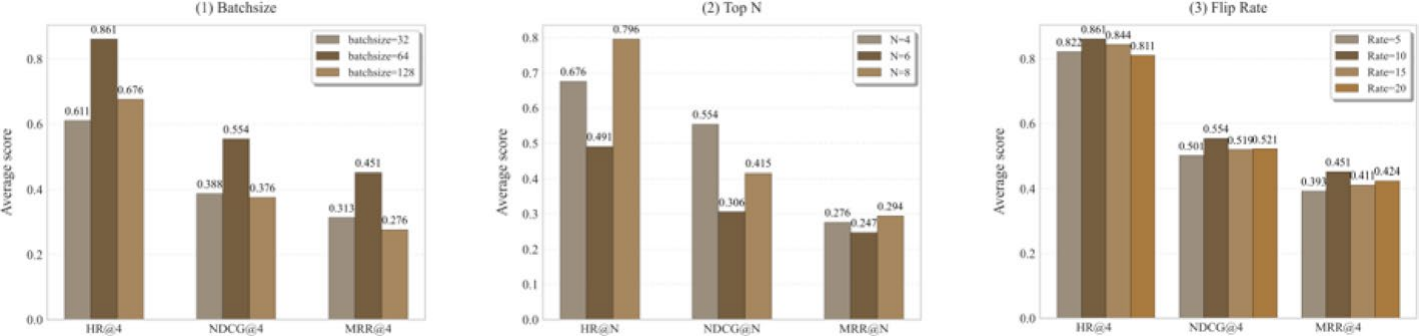
Visualization of parameter sensitivity

Choosing of hyperparameters will affect model performance, under limited page, we mainly discuss top N and rate $\alpha$. When we explore one of them, the others remain fixed to optimal settings. As depicted in Fig. 7(1), as batchsize increases from 32 to 128, model performance tends to increase and then stabilize, reaching the optimum when batch = 64. The effect of risky drug avoidance gradually improves with growing N. However, all metrics drop when *N* = 6. We speculate that it is caused by unstable sampling strategy, and we will strive to improve in subsequent work. Flip rate $\upalpha$ greatly affects data diversity, the MMDDI reaches optimal when $\alpha = 0.1$ as illustrated in Fig. 7(3). This enriches data without excessively damaging key structure information from drug SMILES, thereby improving performance. In terms of overall stability, magnitude is most affected by batchsize due to high memory requirements of MMDDI, while the flip rate has less impact on MMDDI since it increases diversity regardless of its value.    

### Performance in DDI prediction

To further validate the effectiveness of MMDDI in risk assessment, we employed general evaluation metrics (including Accuracy, AUC, F1-score, Precision, Recall, and AUPR) to conduct DDI classification predictions under both transductive and inductive settings. Specifically, risk coefficient threshold is set to 0.5, where drug pairs with value exceeding 0.5 were identified as having potential interactions, while others were classified as not. Three testing configurations were implemented: (S1) Based on DDIs, dataset is partitioned into training and testing sets. In this scenario, the drugs involved in the testing DDIs set are already present in the training set, which is also referred to as the transductive setting; (S2) Dataset partitioning based on drugs, dividing drugs into known and novel drug sets with 4:1, where testing drug pairs set contained one drug belonging to the novel category (i.e., no DDI records associated with this drug existed in the training set); (S3) Employing the same partitioning strategy as S2, but with both drugs in test pairs being novel. Settings S2 and S3 are collectively referred to as inductive settings, as both involve cold-start drugs. Seven advanced models from DDI prediction were selected as baselines, all of which incorporate two or more techniques from multimodal fusion, contrastive learning, graph neural networks, and disentangled representation learning. Validation results on Dataset1 are presented in Table 4, while results for Dataset2 are provided in Supplementary Table S2.

Experimental results demonstrate that MMDDI exhibits outstanding performance across both datasets. In the transductive setting (S1), MMDDI attained 98.42% accuracy and 99.31% AUC, achieving comparable performance to DAS-DDI while significantly outperforming other baseline methods. More importantly, MMDDI maintained excellent performance when simulating cold-start scenarios for new drugs, achieving 98.69% and 93.85% accuracy in S2 and S3, respectively. In contrast to other methods experiencing steep performance declines in inductive settings (e.g., DAS dropping from 99.94% to 75.39% and 50.11%), MMDDI’s stable performance, highlights the advantages of MMDDI’s CL framework. Notably, MMDDI’s performance in S2 even surpassed that in S1. which we hypothesize is attributable to the crucial role of the drug pairs encoder and flip augmentation strategy. The maintained high recall level validates our previous conjecture that additional functional groups may increase the probability of drug interactions. In S3, most baseline metrics dropped to approximately 50% (e.g., PHGL), indicating that methods relying primarily on DDI network construction fail to learn effective representations for novel drugs under strict cold-start conditions. These findings confirm the practical value of the MMDDI framework in providing reliable references for drug safety assessment.

**Table 4:** The performance of MMDDI for DDIs classification prediction on Dataset1

	Model	ACC	AUC	F1	Prec	Rec	AUPR
**S1**	**MDDI-SCL**[13]	93.78	*99.83*	87.55	88.04	87.67	97.82
	**MR-GNN**[55]	69.31	75.44	70.64	67.70	73.85	69.60
	**SSI-DDI**[8]	75.42	83.41	75.04	76.23	73.91	81.42
	**DAS-DDI**[17]	**99.94**	**99.91**	**99.94**	**99.99**	**99.88**	**99.96**
	**SA-DDI**[47]	85.62	92.17	85.93	84.10	87.86	89.60
	**SRR-DDI**[3]	85.15	91.94	85.66	82.86	88.65	89.98
	**PHGL-DDI**[14]	78.40	78.40	80.34	73.65	88.39	76.99
	**MMDDI**	*98.42*	99.31	*98.42*	*98.01*	*98.84*	*99.03*
**S2**	**MDDI-SCL**	67.67	*96.34*	53.04	62.54	48.14	69.47
	**MR-GNN**	62.32	66.84	62.59	62.15	63.05	62.75
	**SSI-DDI**	63.83	69.53	60.53	66.61	55.46	67.49
	**DAS-DDI**	*75.39*	77.01	*67.34*	**99.83**	50.87	*83.25*
	**SA-DDI**	65.91	73.84	57.05	77.04	45.31	73.22
	**SRR-DDI**	63.67	71.37	54.29	73.06	43.28	69.62
	**PHGL-DDI**	54.79	54.79	58.24	54.63	*72.87*	58.15
	**MMDDI**	**98.69**	**99.27**	**98.69**	98.58	**98.81**	**99.07**
**S3**	**MDDI-SCL**	45.89	*90.53*	19.19	25.85	16.78	39.38
	**MR-GNN**	54.42	56.71	*54.32*	54.45	*54.21*	55.01
	**SSI-DDI**	54.87	56.99	44.89	57.77	36.73	56.50
	**DAS-DDI**	50.11	49.40	2.88	58.33	1.49	50.65
	**SA-DDI**	*55.88*	60.13	37.37	*64.44*	26.68	*59.58*
	**SRR-DDI**	53.89	56.41	34.89	59.37	24.72	56.63
	**PHGL-DDI**	50.19	50.19	26.74	34.68	35.92	51.33
	**MMDDI**	**93.85**	**97.21**	**93.96**	**93.00**	**94.96**	**96.36**

### Case study

Taking Fenfluramine (DB00574) as an example, it is one of the CNS drugs used in treating Dravet Syndrome and Lennox-Gastaut Syndrome, once extensively used anti-obesity drug. We anchored Fenfluramine as inhibitor/inducer and assessed potential risk by randomly selecting 128 non-canonical drugs from Dataset1 with which it constituted administration. The top four drugs with risk factor more than 0.5 were Fulvestrant(DB00947), Disopyramide(DB00280), Efonidipine(DB09235), and Clomipramine(DB01242). We validated the output in Dataset2 and found that Fenfluramine enhances CNS depressant effects when combined with Disopyramide. Further, the independent weights of MMDDI outputs for multi-mechanisms decoupling were 0.521 and 0.479, respectively, suggesting that main mechanism of DDI is by altering activity of relevant enzymes and thus affecting the blood drug concentration. As detailed in interaction checker drugs.com [56], Coadministration with fenfluramine may decrease the plasma concentrations and therapeutic efficacy of drugs that are substrates of the CYP450 2B6 and/or CPY3A4 isoenzymes. Meanwhile, Fenfluramine may elevate blood levels of Clomipramine, leading to adverse effects such as drowsiness, blurred vision, constipation. The independent weights of the multi-mechanism were 0.484 and 0.516, respectively. By inhibiting transporters responsible for clomipramine efflux, fenfluramine may cause clomipramine accumulation, thereby increasing the susceptibility to serotonin syndrome.

**Fig. 8:**
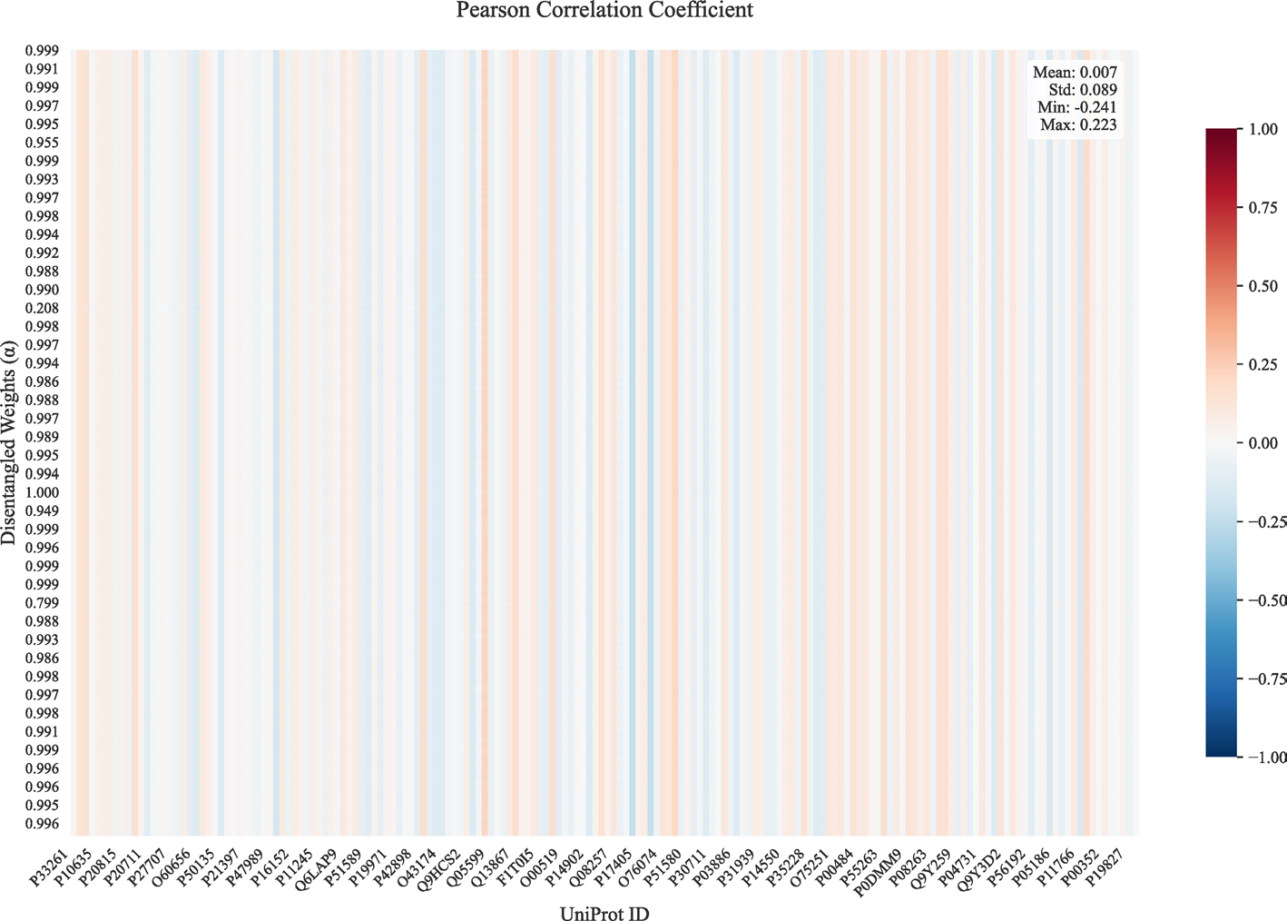
Visualization of decoupled representation and protein enzyme correlations

**Fig. 9:**
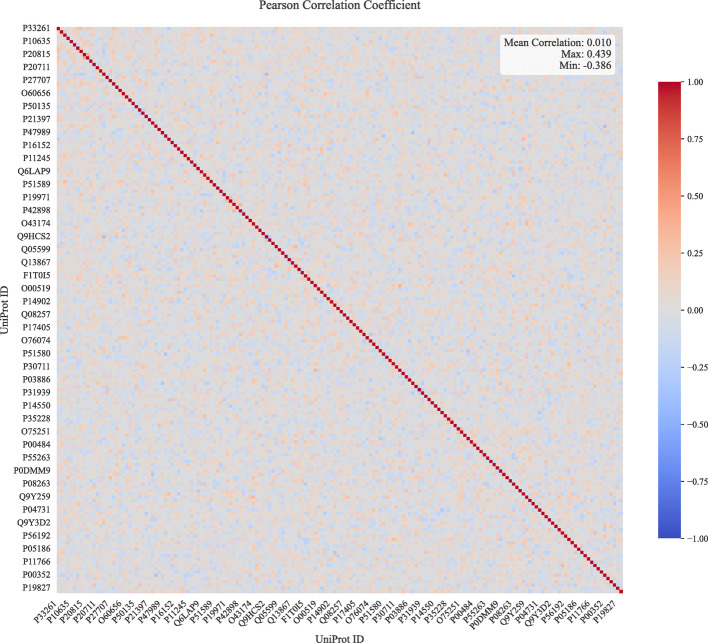
Pearson correlations map of different protein enzyme in latent embedding space

Analysis of Pearson correlations for Dataset 1 (inductive setting S3) reveals key patterns in enzyme-driven DDI mechanisms, as shown in Fig. 8. The *y-axes* correspond to the decoupling weights of different test samples, while the *x-axes*’ UniPro IDs are associated with specific CYP450 enzymes(e.g., P22261 is related to a cytochrome P450 monooxygenase involved in the metabolism of polyunsaturated fatty acids (PUFA)). A small fraction of enzymes contribute to the majority of DDIs, which leads to numerous vertical light-colored stripes in the figure. The correlation value of 0 results from that the corresponding enzymes are not involved in DDIs. The positive or negative nature of the correlation also partially reflects the enzymes’ inductive or inhibitory properties. Simultaneously, low inter-enzyme correlations in the latent space (Fig. 9) confirm the model’s ability to isolate unique features for different enzymes. Supplementary materials provide further evidence of the independence among the disentangled mechanisms.

## Conclusions

In this paper, we devote to tackle data sparsity and mechanism entanglement in co-medication risk identification. As a result, a mechanism-aware co-medication risk evaluator based on CL called MMDDI was proposed. The devised biologically meaningful augmentation strategy overcomes the challenge of limited labeled data, resulting in more robust substructure embeddings. This improvement, validated via ablation studies, is attributable to the multi-view contrastive learning module. Visualization analyses corroborate these findings, showing clear separation and well-defined decision boundaries between positive and negative samples. And the proposed MI constraint decoupling module avoids prediction bias caused by coupling in traditional methods. In the additional DDI prediction task, MMDDI maintains outstanding performance compared to the out of state baselines in inductive setting, achieving accuracy and recall of 0.94 and 0.95, respectively. The case study analyzes the top four drugs in terms of possible risk of co-administration with Fenfluramine, of which both Disopyramide and Clomipramine were validated. The degree of risk aversion of MMDDI for this drug was calculated to be 1.0, 0.63, and 0.5, respectively, and the corresponding independent weights of the MMDDI outputs provide reasonable explanations for the drug’s pathways of action. In addition to MMDDI’s interpretable assistance for co-medication assessment from different perspectives, we believe that it is easily extendable to other tasks such as drug-target interaction risk assessment.

Our experimental design still needs to be improved. In future work, we will tackle several issues to improve accuracy of MMDDI. Toward learning more structural information about drugs, we will consider excavating from 2D and 3D structure of drug. Besides, testing on broader and larger-scale datasets such as Twosides to enable more comprehensive model validation.

## Supplementary Information

Below is the link to the electronic supplementary material.

Supplementary Material 1

Supplementary Material 2

## Data Availability

Two datasets are used in this work. **Dataset1**[10]: The first data set can be downloaded from https://github.com/ShenggengLin/MDF-SA-DDI/blob/main/event.zip. **Dataset2**[22]:The second data set is available from https://github.com/ShenggengLin/MDF-SA-DDI/blob/main/Dataset2_drug_information.zip to download. Interaction checker **Drugs.com**: https://www.drugs.com/. Chemical database **PubChem**: https://pubchem.ncbi.nlm.nih.gov/. The main code can be accessed at https://github.com/YunjvZeng/MMDDI, we will further organize and refine it later.

## Abbreviations

DDI: Drug-Drug Interactions
MMDDI: Multi-Mechanism Disentangled Drug-drug Interaction assessment framework
ADR: Adverse drug reactions
NLP: Natural language processing
SMILES: Simplified molecular input line entry system
GAT: Graph attention network
CL: Contrastive learning
ADMET: Absorption, distribution, metabolism, excretion, and toxicity
AE: Auto encoder
MHA: Multi head attention
ACC: Accuracy
HR: Hit rate
NDCG: Normalized discounted cumulative gain
MRR: Mean reciprocal rank
BGD: Batch gradient descent
