# Supplementary material for: Contrastive learning-based multi-mechanism disentangled assessment for drug-drug interaction
Source: BMC Bioinformatics. 2025 Nov 27;26:286. doi: 10.1186/s12859-025-06304-z (PMC12659097; doi:10.1186/s12859-025-06304-z)
Supplement: Supplementary file 1 — Supplementary Material 1 [file 12859_2025_6304_MOESM1_ESM.docx]

**Additional Experiments**

***Performance in DDI prediction***

To further validate the effectiveness of MMDDI in risk assessment, we employed general evaluation metrics (including Accuracy, AUC, F1-score, Precision, Recall, and AUPR) to conduct DDI classification predictions under both transductive and inductive settings. Specifically, risk coefficient threshold is set to 0.5, where drug pairs with value exceeding 0.5 were identified as having potential interactions, while others were classified as not. Three testing configurations were implemented: (S1) Based on DDIs, dataset is partitioned into training and testing sets. In this scenario, the drugs involved in the testing DDIs set are already present in the training set, which is also referred to as the transductive setting; (S2) Dataset partitioning based on drugs, dividing drugs into known and novel drug sets with 4:1, where testing drug pairs set contained one drug belonging to the novel category (i.e., no DDI records associated with this drug existed in the training set); (S3) Employing the same partitioning strategy as S2, but with both drugs in test pairs being novel. Settings S2 and S3 are collectively referred to as inductive settings, as both involve cold-start drugs. Seven advanced models from DDI prediction were selected as baselines, all of which incorporate two or more techniques from multimodal fusion, contrastive learning, graph neural networks, and disentangled representation learning. Validation results on Dataset1 are presented in Table 4, while results for Dataset2 are provided in Supplementary Table S2.

Experimental results demonstrate that MMDDI exhibits outstanding performance across both datasets. In the transductive setting (S1), MMDDI attained 98.42% accuracy and 99.31% AUC, achieving comparable performance to DAS-DDI while significantly outperforming other baseline methods. More importantly, MMDDI maintained excellent performance when simulating cold-start scenarios for new drugs, achieving 98.69% and 93.85% accuracy in S2 and S3, respectively. In contrast to other methods experiencing steep performance declines in inductive settings (e.g., DAS dropping from 99.94% to 75.39% and 50.11%), MMDDI's stable performance, highlights the advantages of MMDDI's CL framework. Notably, MMDDI's performance in S2 even surpassed that in S1. which we hypothesize is attributable to the crucial role of the drug pairs encoder and flip augmentation strategy. The maintained high recall level validates our previous conjecture that additional functional groups may increase the probability of drug interactions. In S3, most baseline metrics dropped to approximately 50% (e.g., PHGL), indicating that methods relying primarily on DDI network construction fail to learn effective representations for novel drugs under strict cold-start conditions. These findings confirm the practical value of the MMDDI framework in providing reliable references for drug safety assessment.

Table S1: The performance of MMDDI for DDIs classification prediction on Dataset1.

|  | **Model** | **ACC** | **AUC** | **F1** | **Prec** | **Rec** | **AUPR** |
| --- | --- | --- | --- | --- | --- | --- | --- |
| **S1** | **MDDI-SCL** | 93.78 | 99.83 | 87.55 | 88.04 | 87.67 | 97.82 |
|  | **MR-GNN** | 69.31 | 75.44 | 70.64 | 67.70 | 73.85 | 69.60 |
|  | **SSI-DDI** | 75.42 | 83.41 | 75.04 | 76.23 | 73.91 | 81.42 |
|  | **DAS-DDI** | **99.94** | **99.91** | **99.94** | **99.99** | **99.88** | **99.96** |
|  | **SA-DDI** | 85.62 | 92.17 | 85.93 | 84.10 | 87.86 | 89.60 |
|  | **SRR-DDI** | 85.15 | 91.94 | 85.66 | 82.86 | 88.65 | 89.98 |
|  | **PHGL-DDI** | 78.40 | 78.40 | 80.34 | 73.65 | 88.39 | 76.99 |
|  | **MMDDI** | 98.42 | 99.31 | 98.42 | 98.01 | 98.84 | 99.03 |
| **S2** | **MDDI-SCL** | 67.67 | 96.34 | 53.04 | 62.54 | 48.14 | 69.47 |
|  | **MR-GNN** | 62.32 | 66.84 | 62.59 | 62.15 | 63.05 | 62.75 |
|  | **SSI-DDI** | 63.83 | 69.53 | 60.53 | 66.61 | 55.46 | 67.49 |
|  | **DAS-DDI** | 75.39 | 77.01 | 67.34 | **99.83** | 50.87 | 83.25 |
|  | **SA-DDI** | 65.91 | 73.84 | 57.05 | 77.04 | 45.31 | 73.22 |
|  | **SRR-DDI** | 63.67 | 71.37 | 54.29 | 73.06 | 43.28 | 69.62 |
|  | **PHGL-DDI** | 54.79 | 54.79 | 58.24 | 54.63 | 72.87 | 58.15 |
|  | **MMDDI** | **98.69** | **99.27** | **98.69** | 98.58 | **98.81** | **99.07** |
| **S3** | **MDDI-SCL** | 45.89 | 90.53 | 19.19 | 25.85 | 16.78 | 39.38 |
|  | **MR-GNN** | 54.42 | 56.71 | 54.32 | 54.45 | 54.21 | 55.01 |
|  | **SSI-DDI** | 54.87 | 56.99 | 44.89 | 57.77 | 36.73 | 56.50 |
|  | **DAS-DDI** | 50.11 | 49.40 | 2.88 | 58.33 | 1.49 | 50.65 |
|  | **SA-DDI** | 55.88 | 60.13 | 37.37 | 64.44 | 26.68 | 59.58 |
|  | **SRR-DDI** | 53.89 | 56.41 | 34.89 | 59.37 | 24.72 | 56.63 |
|  | **PHGL-DDI** | 50.19 | 50.19 | 26.74 | 34.68 | 35.92 | 51.33 |
|  | **MMDDI** | **93.85** | **97.21** | **93.96** | **93.00** | **94.96** | **96.36** |

Table S2: The performance of MMDDI for DDIs classification prediction on Dataset2.

|  | **Model** | **ACC** | **AUC** | **F1** | **Prec** | **Rec** | **AUPR** |
| --- | --- | --- | --- | --- | --- | --- | --- |
| **S1** | **MDDI-SCL** | 95.16 | 99.95 | 93.21 | 91.62 | 95.00 | 98.62 |
|  | **MR-GNN** | 65.68 | 70.78 | 68.69 | 63.15 | 75.29 | 65.11 |
|  | **SSI-DDI** | 76.76 | 85.12 | 77.16 | 75.83 | 78.54 | 83.42 |
|  | **DAS-DDI** | **99.99** | **100.00** | **99.99** | **99.98** | **99.99** | **100.00** |
|  | **SA-DDI** | 92.37 | 97.33 | 92.50 | 90.93 | 94.13 | 96.71 |
|  | **SRR-DDI** | 92.19 | 96.64 | 92.34 | 90.53 | 94.23 | 95.67 |
|  | **PHGL-DDI** | 70.61 | 70.61 | 74.53 | 65.78 | 86.00 | 68.48 |
|  | **MMDDI** | 99.69 | 99.84 | 99.69 | 99.80 | 99.58 | 99.81 |
| **S2** | **MDDI-SCL** | 65.95 | 97.57 | 55.78 | 56.05 | 57.12 | 67.94 |
|  | **MR-GNN** | 60.31 | 63.73 | 63.33 | 58.86 | 68.53 | 59.27 |
|  | **SSI-DDI** | 66.89 | 73.96 | 62.71 | 71.78 | 55.69 | 73.27 |
|  | **DAS-DDI** | 75.54 | 83.92 | 67.66 | **99.81** | 51.18 | 88.62 |
|  | **SA-DDI** | 68.95 | 77.39 | 61.16 | 81.54 | 48.97 | 77.83 |
|  | **SRR-DDI** | 68.52 | 76.38 | 61.50 | 79.14 | 50.37 | 76.07 |
|  | **PHGL-DDI** | 48.74 | 48.74 | 41.41 | 49.05 | 46.87 | 49.78 |
|  | **MMDDI** | **99.82** | **99.87** | **99.82** | 99.80 | **99.83** | **99.80** |
| **S3** | **MDDI-SCL** | 46.96 | 93.15 | 28.38 | 31.60 | 27.73 | 42.61 |
|  | **MR-GNN** | 54.82 | 56.31 | 57.64 | 54.25 | 61.52 | 54.18 |
|  | **SSI-DDI** | 58.56 | 63.50 | 48.73 | 63.89 | 39.39 | 62.71 |
|  | **DAS-DDI** | 50.04 | 77.10 | 1.00 | 42.59 | 0.51 | 77.66 |
|  | **SA-DDI** | 56.02 | 61.11 | 34.19 | 67.96 | 22.86 | 61.10 |
|  | **SRR-DDI** | 57.45 | 62.15 | 46.70 | 64.42 | 39.57 | 61.10 |
|  | **PHGL-DDI** | 49.96 | 49.96 | 22.49 | 29.71 | 33.47 | 49.69 |
|  | **MMDDI** | **96.67** | **97.02** | **96.69** | **95.96** | **97.44** | **95.48** |

***Visualization for case Study***


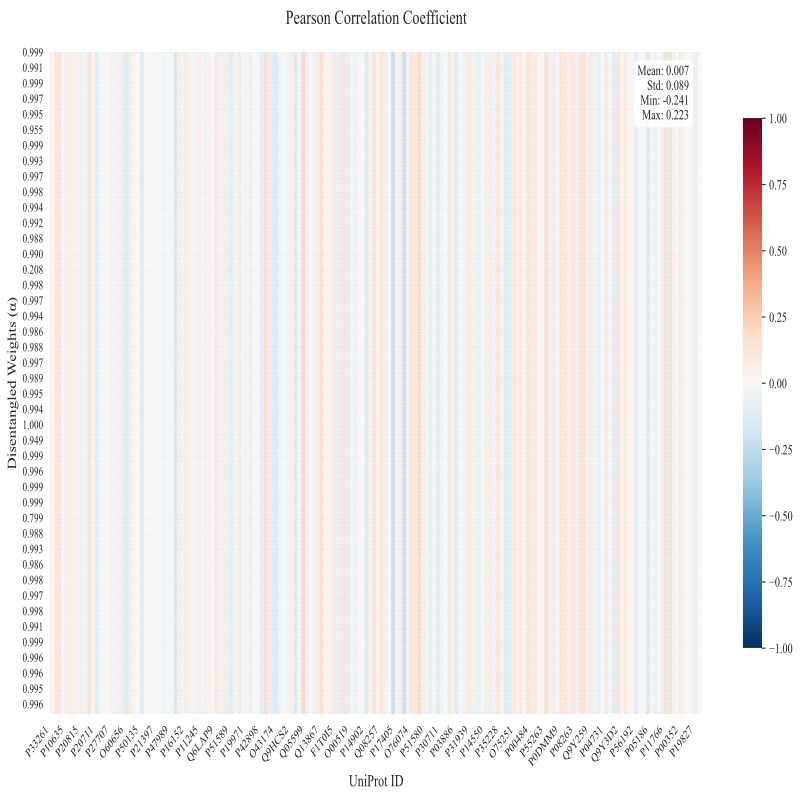


Fig. S1 Visualization of decoupled representation and protein enzyme correlations.


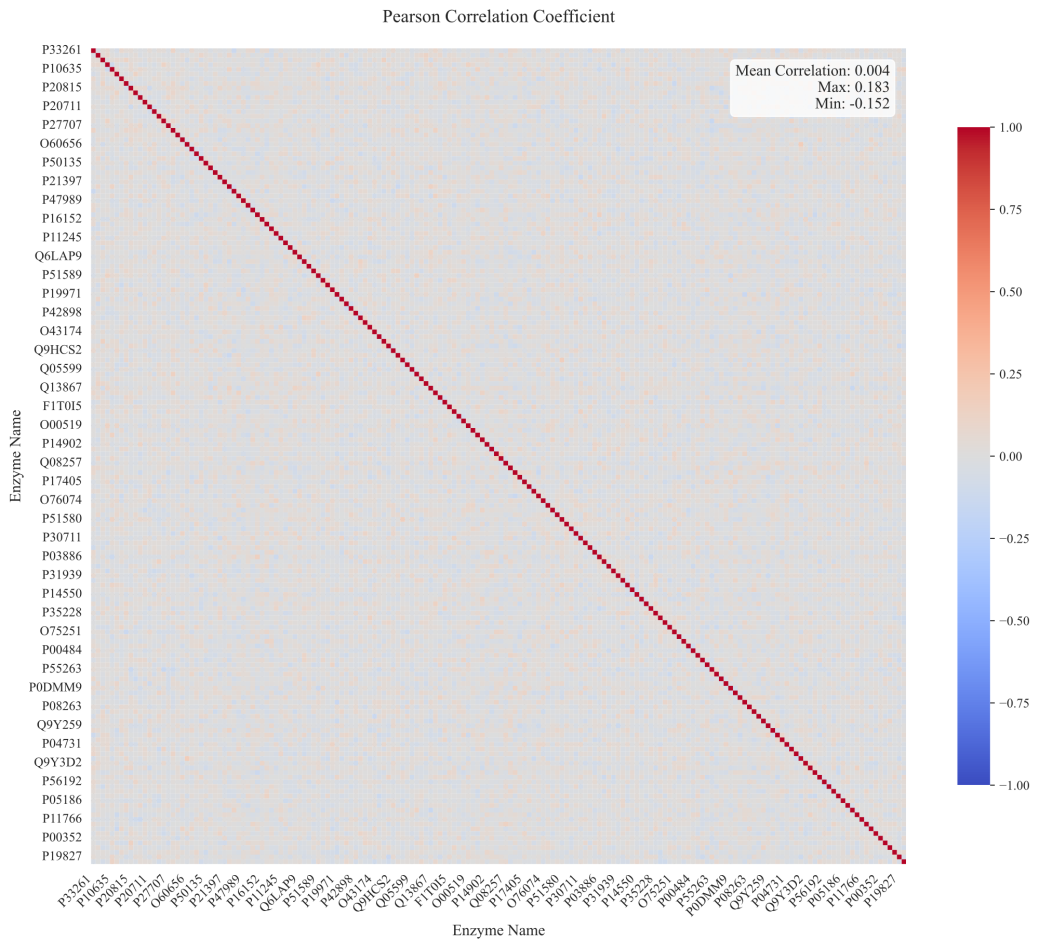


Fig. S2 Pearson correlations map of different protein enzyme in latent embedding space.


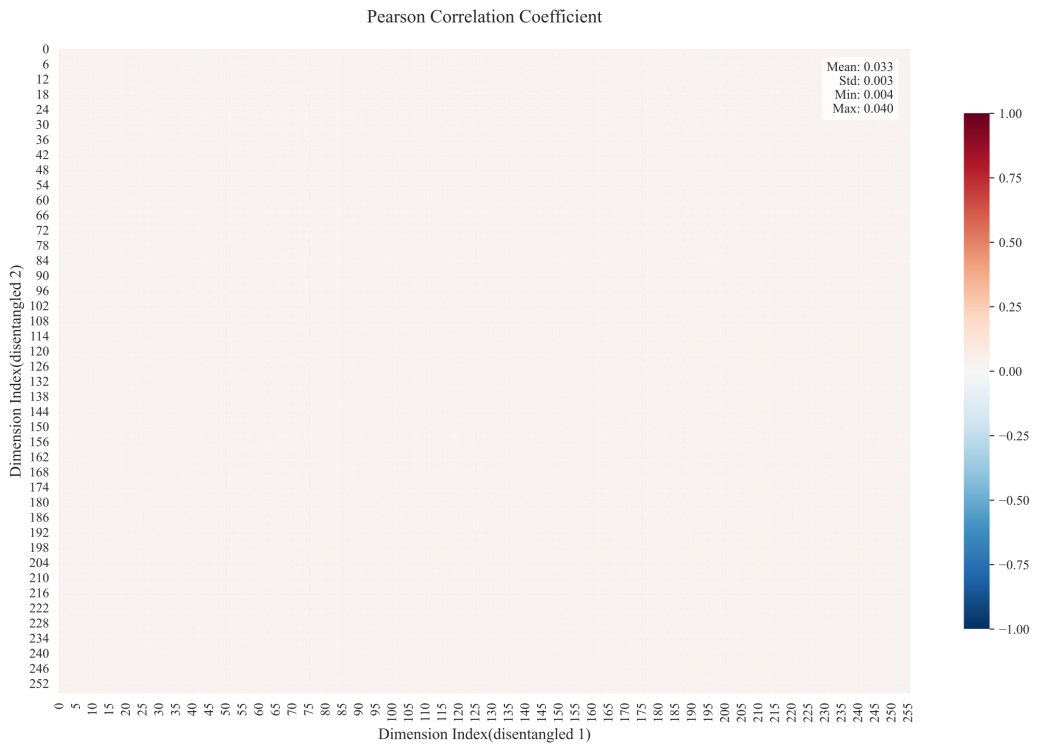


Fig. S3 Pearson correlations map of disentangled mechanism representation on 256 testing DDIs.
